# Profiling the Anti‐Photoaging Impact of Titanium Dioxide and Zinc Oxide Nanoparticles: A Focus on Signaling Pathways

**DOI:** 10.1096/fj.202500342R

**Published:** 2025-04-28

**Authors:** Neil Dominic T Pangilinan, Mohammad Shalbaf, Aline Souza, Bhaven Chavan, Catherine Bonn, Mark A Birch‐Machin

**Affiliations:** ^1^ Dermatological Sciences, Translational and Clinical Research Institute Newcastle University Newcastle upon Tyne Tyne and Wear UK; ^2^ Croda Europe Ltd Snaith UK

**Keywords:** photoaging, photoprotection, titanium dioxide, ultraviolet rays, zinc oxide

## Abstract

Inorganic nanoparticles are known to protect skin from ultraviolet rays (UVR) and delay photoaging. However, the photoprotective effects of these nanoparticles have not been broadly analyzed at a genetic level. The study objectives are as follows: (1) to investigate how UV‐only and complete solar light can affect signaling pathways and genes related to photoaging in human dermal fibroblasts; (2) to investigate how TiO_2_ and ZnO nanoparticles provide photoprotection at a genetic level. RNAseq identified pathways and genes that were significantly affected by both irradiation conditions. Extracellular matrix (ECM) remodeling, inflammation, and cell cycle‐related genes were subsequently validated by qPCR. The photoprotective properties of < 100 nm TiO_2_ and ZnO dispersions at a 25% active level were analyzed through quantitative differences in the irradiation‐induced expression of these genes. There were < 15 signaling pathways affected by UV and complete solar light (*p*‐value (−log10) > 1). Significant differences in gene expression following irradiation were found in MMP1, MMP3, PTGS1, PTGES, MDM2, CDKN1A, and CCNE2 (*p* ≤ 0.05) through qPCR. TiO_2_ and ZnO minimized the irradiation‐induced expression of genes involved in the inhibition of matrix metalloproteinases, prostanoid biosynthesis, and cell cycle pathways. Photoprotection was best observed in cell cycle‐related genes, showing expression differences of up to 74% (*p* ≤ 0.0001). However, no distinct differences in photoprotection between TiO_2_ and ZnO were found. The findings from this study serve as a framework for future optimization and development of inorganic sunscreen formulations to target genes that contribute to different aspects of skin aging.

## Graphical abstract

UV wavelengths from solar light trigger signaling pathways in dermal fibroblasts that accelerate photoaging. RNAseq analysis revealed that signaling pathways relating to the cell cycle, ECM remodeling, and inflammation were significantly activated or inactivated 24 h post‐irradiation. Titanium dioxide and zinc oxide inorganic UV filters < 100 nm were effective in alleviating irradiation‐induced upregulation or downregulation in multiple genes within these pathways. Our study provides novel insights on how inorganic‐based photoprotection has an anti‐photoaging impact on cell signaling and gene activity.

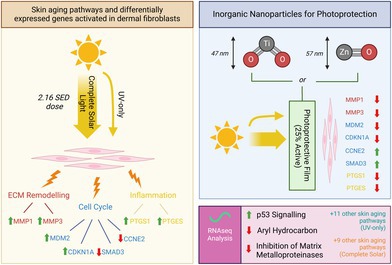

## 1 Introduction

The development of sunscreen interventions has seen an increasing demand in the market over the last few decades due to increasing research and awareness on photoaging. By the end of 2024, it is estimated that the global sales for sunscreens will reach $10.7 billion [1]. Sunscreens, which are accessible to the public through retail establishments or prescriptions as creams, are formulated to minimize the harmful effects of ultraviolet rays (UVR) from the sun [2]. Photodamage, as a consequence of UVR exposure, can be defined as solar light‐induced deoxyribonucleic acid (DNA) damage, which can speed up the intrinsic aging process and give rise to photoaged skin. DNA damage can occur directly or indirectly through the release of reactive oxygen species (ROS) in the mitochondria and oxidative stress [3].

Ultraviolet radiation (UVR), characterized by high energy and short wavelength, can be split into two types which reach the earth's surface: ultraviolet A (UVA, 315–400 nm) and ultraviolet B (UVB, 280–315 nm). The composition of UVR received at the Earth's surface is 95% UVA and 5% UVB; this is equivalent to 5% and 0.1% of the total solar light received, respectively [4, 5]. UVR is known to be the most dangerous component of solar light and can cause acute or chronic effects on the skin. Acute symptoms of UVR exposure include changes in pigmentation, erythema (sunburn), and tanning [6, 7], while chronic symptoms include increased skin malignancy and photoaging [8, 9]. It is reported that 80% of visible face aging in Caucasians is attributed to exposure to UVR [8]. These observable effects can be attributed to the UVA and/or UVB components of UVR, which penetrate skin differently.

UVA can affect both the epidermis and the dermis, while UVB is mostly absorbed by the epidermis [10]. Studies on human skin in vitro and in vivo have consistently reported a decrease in collagen levels within the dermis due to an increase in collagenase production upon acute exposure to UVA; the most prominent of which are MMP‐1 and MMP‐9 [11, 12, 13]. These collagenases break down the existing collagen fibers, which provide stability to the skin and thus accelerate symptoms of photo‐aging. Additionally, multiple studies on human skin have confirmed a link between UVA exposure and the onset of elastosis, which is characterized as thick, wrinkly skin [14, 15]. Unlike UVA, UVB is a shorter wavelength and higher energy radiation, which can cause direct damage to DNA. Although UVB can contribute to photoaging, it is mostly associated with the progression of skin cancers, which can be split into two types: melanomas and non‐melanomas. It is reported that 86% of skin cancers are sourced from exposure to UVB [16].

An international quantification parameter for UVR exposure is the standard erythemal dose (SED) which weighs UV wavelengths based on their ability to cause erythema, regardless of skin type and sensitivity [17]. The dose for 1 SED is defined as 10 mJ.cm^−2^ (100 J.m^−2^). A reference action spectrum by McKinlay and Diffey showed that an SED of 2.0–2.5 led to mild reddening in fair‐skinned individuals 24 h after UVR exposure. Irradiation studies can use this range as a basis for the dose required to induce physiological changes in cells. UV light, independently or as combined solar light, has previously been found to upregulate damage biomarkers in human dermal fibroblasts 24 h after a 2.16 SED dose [18].

Due to the photoaging and skin cancer stimulating effects of UVR, the application of sunscreens in everyday life is heavily promoted. Daily and chronic use of these products has been reported to minimize the rate of melanoma progression and reduce signs of photoaging such as solar elastosis [19, 20]. However, the variety of sunscreens is broad due to differences in active ingredients and different mechanisms of protection.

Physical sunscreens are a classification of sunscreens which contain inorganic active ingredients and provide protection by reflecting harmful wavelengths of light. The most common active ingredients are titanium dioxide (TiO_2_) and zinc oxide (ZnO). Although traditionally known to reflect UVR, recent evidence has shown that they also absorb UVR [21]. Both filters provide broad‐spectrum protection; in particular, TiO_2_ is understood to be more effective at filtering UVB while ZnO efficiently targets UVA [22]. When combining TiO_2_ and ZnO particles, it is reported that there is a broader range of photoprotection against UVB and UVA [23]. These active ingredients now exist as nanoparticles, with a defined size < 100 nm, which reduce the opaqueness of the sunscreen and improve the cosmetic properties desired by users [24].

This paper aims to understand how TiO_2_ and ZnO nanoparticles provide photoprotection for dermal fibroblasts at a genetic level, through their effect on signaling pathways and genes related to photoaging. Relevant signaling pathways and associated genes which are differentially expressed 24 h post‐irradiation with complete solar and UV‐only are firstly identified using RNAseq. Then, a variety of genes relating to extracellular matrix (ECM) remodeling, inflammation, and cell cycle are validated by qPCR. Lastly, the photoprotective properties of TiO_2_ and ZnO dispersions are analyzed through quantitative differences in the irradiation‐induced expression of the chosen genes. The outcomes of this research would be beneficial for future genetic studies related to photoaging and the development of new sunscreen formulations.

## 2 Materials and Methods

### 2.1 RNAseq Analyses

RNAseq was performed on RNA obtained from neonatal human dermal fibroblasts (HDFn) 24 h post‐irradiation with 2.16 SED complete solar light or UV‐only light. On RStudio, sequences were pseudo‐aligned using Salmon and differentially expressed genes were determined using DESeq2.

#### 2.1.1 Preliminary DEG Analysis

RStudio, in tandem with the pseudo‐aligned RNAseq data, was used to generate volcano plots, which visualize the distribution of differentially expressed genes (DEGs) in terms of log fold change against log adjusted *p*‐value. A Venn diagram was also created to identify the relationship between DEGs in different irradiation conditions. RNAseq data from HDFn irradiated with complete visible light (~400–700 nm) was used as a control to further distinguish the impact of UV wavelengths on DEGs. The thresholds applied on RStudio to filter the number of DEGs were as follows: adjusted *p*‐value < 0.05, and log_2_ fold change < −0.5 and > 0.5.

#### 2.1.2 Pathway Analysis

Pathway analysis was conducted for UV‐only and complete solar conditions using Ingenuity Pathway Analysis (IPA) software (Qiagen, Germany). TSV files were processed using the IPA software with the following thresholds: adjusted *p*‐value < 0.05 and log_2_ fold change < −0.5 and > 0.5. The thresholds filtered out pathways that were minimally affected by irradiation. For each condition, a list of signaling pathways ordered by *p*‐value (−log_10_) was printed by the software. Pathways relevant to skin aging that appeared in the pathway lists for either irradiation condition were identified from the software based on aspects such as cell cycle regulation, immune response, wound healing, and biosynthesis of specific molecules. A pathway was determined to be significant if *p*‐value (−log10) > 1, as this value further excluded a large number of pathways.

#### 2.1.3 Analysis of DEGs Within Signaling Pathways

The differentially expressed gene (DEG) lists for each pathway for each condition were printed from the IPA software. The DEGs were organized in descending order of experimental log ratio. A number of genes were chosen for further analysis based on two or more of the following criteria: (1) high or low experimental log ratio, (2) differential expression in both irradiation conditions, and (3) found in multiple pathways.

### 2.2 Cell Culture

#### 2.2.1 Maintenance of HDFns

HDFn (Invitrogen, UK), between passages 10–20, were cultured in 2D monolayers and maintained in standard DMEM (DMEM, ThermoFisher Scientific, USA, contains L‐glutamine, phenol red, sodium pyruvate, and sodium bicarbonate) supplemented with 1% penicillin/streptomycin and 10% FBS in T175 flasks; this can be referred to as complete DMEM. The cells were incubated at 37°C/5% carbon dioxide (CO_2_) until they were passaged or seeded for experiments. Passaging of cells was undertaken twice a week when confluency was 70%–90%.

#### 2.2.2 Cell Seeding

HDFn were seeded into 35 mm TPP dishes (Corning, USA) at a cell density of 35,000 cells per mL. A Countess 3 Automated Cell Counter (Invitrogen, USA) was used to aid cell seeding calculations, as per the manufacturer's instructions. All TPP plates used for irradiation studies were sealed with parafilm and incubated for 24 h at 37°C/5% CO_2_ in 2 mL complete DMEM prior to irradiation.

### 2.3 Preparation of Inorganic UV Filters

47 nm TiO_2_ and 57 nm ZnO in dispersions were diluted with medium‐chain triglyceride oil to acquire dispersions with a 25% active level and homogenized. The particle sizes defined are mean values acquired upon testing with X‐ray diffraction crystallography (XRDC). Following European regulations, TiO_2_ used in this study, in its rutile form, was coated with alumina stearate, a base layer aimed at reducing its photoactivity [25]. ZnO used in this study was uncoated and dispersed in an oil carrier using polymeric dispersants, which create polymer‐induced interactions and enable the particles to be in situ coated [26]. Thin films of TiO_2_ and/or ZnO to use as a photoprotective barrier between the light source and sample during irradiation were prepared using the Transpore tape approach established by Diffey and Robson [27] and highly reproduced in various UV irradiation studies [28, 29]. Translucent Transpore tape (3 M, USA) was cut into 5 cm × 5.3 cm squares, with a surface area that covered the top of the 35 mm TPP cell culture dishes. Using a tared scale, 2 mg/cm^2^ of each dispersion was applied onto the Transpore tape and evenly spread across its surface. The dispersions were left to dry on the tape at room temperature for half an hour to ensure that a consistent film developed on the tape.

### 2.4 Irradiation With the Solar Simulator

Complete solar light (280–1100 nm) and UV‐only (280–410 nm) irradiation was conducted using a Newport Solar Simulator (Newport Oriel model 91 292, 1000 W, MKS Instruments Inc., USA). The output of solar light from the Solar Simulator was compared to the sun's output at solar noon in midsummer in the Mediterranean at 45° latitude, as calculated by Diffey [30]. The dose used for irradiation studies was 2.16 SED, which is equivalent to 20 min in the condition above. Previous calibrations of the Solar Simulator using a FLAME‐S‐XR1‐ES spectroradiometer with CC‐3‐DA direct‐attach cosine corrector and spectralon diffuser (Ocean Optics, the Netherlands) enabled calculation of a 2.16 SED dose at a consistent distance from the lamp, regardless of daily variations in the lamp's output.

Prior to irradiation, the Solar Simulator was left to warm up for 15 min, and a measurement was taken with a radiometer (model IL1400A, serial 8524 with UVA sensor, serial 867, International Light Technologies, USA) to account for changes in daily lamp output. The time of irradiation would subsequently be calculated to deliver a 2.16 SED dose. The complete solar condition did not require the use of any light filters, while UV‐only required an IR/VIS filter.

Fibroblasts were irradiated in phenol red‐free and FBS‐free clear DMEM (DMEM, ThermoFisher Scientific, USA, does not contain L‐glutamine, phenol red, and sodium pyruvate) in 35 mm TPP plates, and plastic lids were removed as they are known UV absorbers. For photoprotected samples, Transpore tape with a film of inorganic UV filter was suspended in air on the rim of the TPP dishes between the light source and cells; the tape was not in direct contact with the media and HDFns.

### 2.5 RNA Extraction and cDNA Synthesis

HDFn were washed with phosphate buffered saline (PBS) and detached from the dishes using trypsin–EDTA (TE), 24 h after irradiation. Complete DMEM was added to neutralize TE, and the cells were centrifuged at 300 ***g*** for 5 min to produce a cell pellet. The media was aspirated and replaced with 350 μL of RLT lysis buffer (included in the RNEasy mini kit (Qiagen, UK)) supplemented with 10 μL/mL β‐mercaptoethanol (ThermoFisher Scientific, USA). Total RNA was collected using the RNeasy mini kit, following the manufacturer's instructions. Total RNA concentration was measured using a NanoDrop ND‐100 (Nanodrop Technologies LLC, ThermoFisher Scientific, USA) and RNA purity was assessed using the 260/280 nm ratio provided by the NanoDrop. The RNA samples were converted to complementary DNA (cDNA) by reverse transcription using the High Capacity cDNA Reverse Transcription Kit (ThermoFisher Scientific, USA) with RNAse inhibitor (ThermoFisher Scientific, USA), as per the manufacturer's instructions.

### 2.6 Relative Quantification of Gene Expression With TaqMan qPCR

Quantitative polymerase chain reaction (qPCR) was performed on cDNA at a standardized concentration of 5 ng/μL using Applied Biosystems QuantStudio3 (ThermoFisher Scientific, USA). The plate plan and qPCR settings were pre‐set using the Design and Analysis Software on ThermoFisher Cloud. The primers used were Taqman Gene Expression Assays, which had a FAM‐MGB dye label for detection. The TaqMan gene expression assay primers used for qPCR are shown in Table 1.

**TABLE 1:** TaqMan gene expression assay primers used in qPCR analysis of UV and complete solar light irradiated human dermal fibroblasts. Target species for all primers is human.

Target	Dye	Code	Target	Dye	Code
CCNE2	FAM	Hs00180319_m1	MMP3	FAM	Hs00968305_m1
CDKN1A	FAM	Hs00355782_m1	PTGES	FAM	Hs00610420_m1
GAPDH	FAM	Hs02786624_g1	PTGS1	FAM	Hs00377726_m1
MDM2	FAM	Hs00540450_s1	SMAD3	FAM	Hs00969210_m1
MMP1	FAM	Hs00899658_m1			

The housekeeping gene used for all experiments was glyceraldehyde 3‐phosphate dehydrogenase (GAPDH). One reaction mixture consisted of 1 μL of 20 × TaqMan gene expression assay, 10 μL 2 × TaqMan Fast Advanced Master Mix (4 369 016, ThermoFisher Scientific, USA), 4 μL of 5 ng/μL DNA in RNase free water, and 5 μL of RNase free water (20 μL total).

For each qPCR run, technical repeats were carried out in triplicate. The run was also repeated an additional two times to acquire three biological repeats. Upon acquiring both technical and biological repeats, mean *C*_t_ values were calculated. Fold change was calculated using the ΔΔ*C*_t_ method, which culminated in fold change being observed as $2^{-\Delta \Delta C_{t}}$. The cut off for *C*_t_ values was 35 cycles. For analysis, the $2^{-\Delta \Delta C_{t}}$ fold change values were converted to log_2_ values to clearly identify the upregulation and downregulation patterns in gene expression with respect to the non‐irradiated controls.

### 2.7 Statistical Analysis

The statistical significance of quantitative results was assessed by applying relevant statistical tests on GraphPad Prism 9. For this study, one‐way ANOVA was used on datasets, where applicable, with Tukey's post hoc test. The statistical differences of a dataset are graphically represented as: ns = *p* > 0.05, *p < = 0.05, **p < = 0.01, ***p < = 0.001, ****p < = 0.0001.

## 3 Results

### 3.1 Signaling Pathways and DEGs Involved in ECM Remodeling, Inflammation, and Cell Cycle Were Induced by UV Light

**FIGURE 1:**
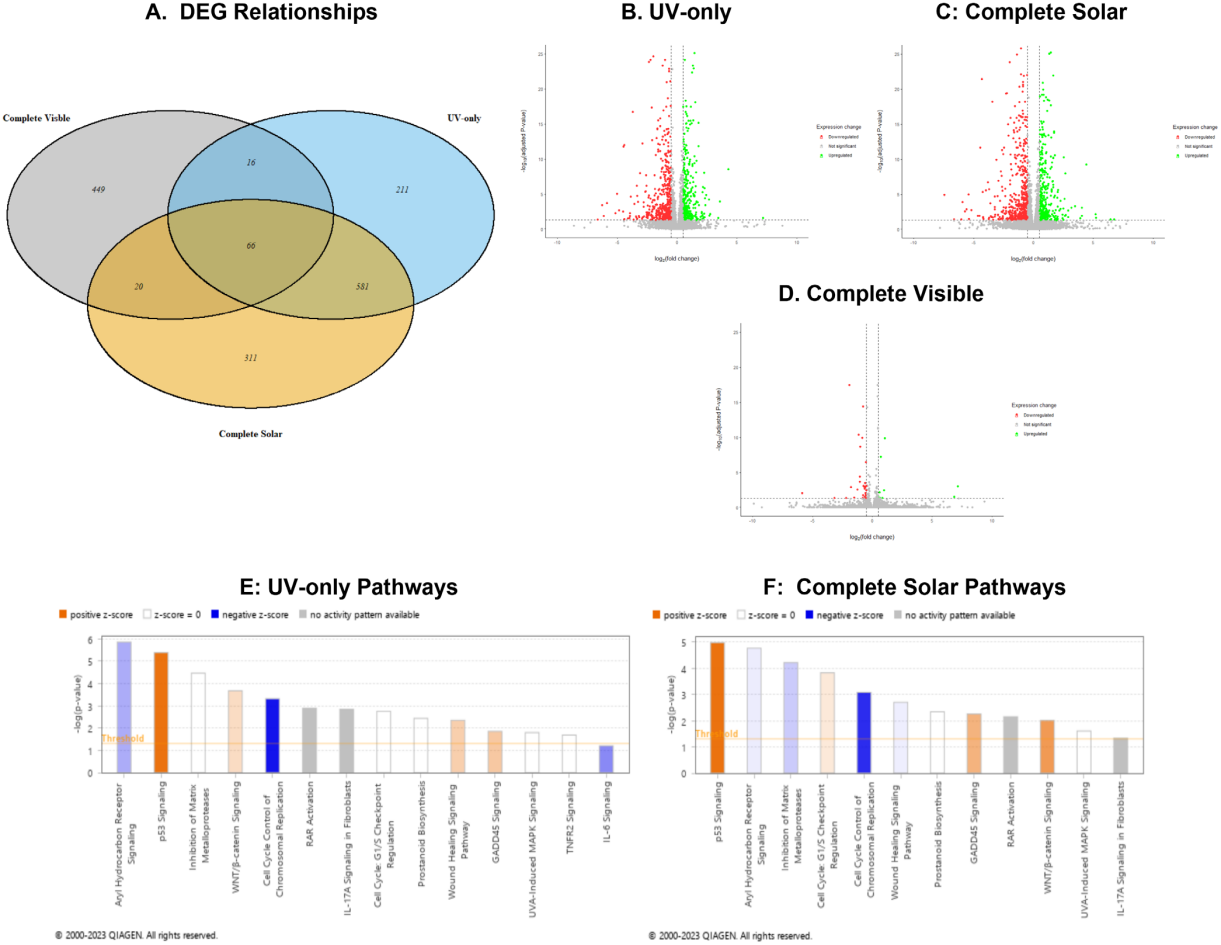
RNAseq Analyses to identify signaling pathways and differentially expressed genes (DEGs) in human dermal fibroblasts 24 h post‐irradiation with UV‐only and complete solar light. Irradiation with complete visible light (~400–700 nm) was used as a control. (A) Venn diagram showing the relationship of DEGs between the three irradiation conditions. (B) Volcano plot to show the distribution of DEGs in UV‐only; (C) Complete solar; (D) and Complete Visible. (E) Signaling pathways affected by UV‐only (−log(*p*‐value) > 1); (B) Pathways affected by complete solar light (−log(*p*‐value) > 1). Positive z‐score = pathway activation. Negative z‐score = pathway inhibition. Z score = 0 = pathway may be activated or inhibited. Threshold −log(*p*‐value) suggested by IPA = 1.3. *N* = 3.

Preliminary RNAseq analyses on RStudio showed that 874 genes were differentially expressed following UV‐only irradiation, 978 following complete solar, and 551 following complete visible (Figure 1A). There were 683 DEGs shared between two or more conditions, with 581 being shared exclusively between UV‐only and complete solar. These values suggest that UV wavelengths are a major contributor to changes in gene expression compared to wavelengths of the visible spectrum. Additionally, the volcano plots for UV‐only and complete solar (Figure 1B,C) show similarity in the frequency and distribution of DEGs when plotting log adjusted *p*‐value against log fold change, with the thresholds in Section 2.1.1 applied. The volcano plot for complete visible light (Figure 1D) contrarily shows that very few DEGs pass the threshold for adjusted *p*‐value (> 1). Therefore, it is implied that DEG frequency and distribution for complete solar is primarily caused by UV wavelengths.

The IPA software identified over 180 signaling pathways that were affected by irradiation with UV‐only and/or complete solar light. The majority of these pathways satisfied the log ratio and *p*‐value thresholds stated prior to pathway analysis and were printed out by the software. UV‐only and complete solar light irradiation induced significant changes in > 10 skin‐related pathways, with the former affecting 2 extra pathways when *p*‐value (−log10) > 1 (Figure 1E,F). Additionally, these two conditions share a similar profile whereby the p53 signaling pathway, aryl hydrocarbon receptor signaling, and inhibition of matrix metalloproteinases are the most affected pathways (−log (*p*‐value) > 4).

The z‐scores in Figure 1 predict the chance of a pathway being activated or inactivated. The p53 pathway is strongly activated in the presence of UV (Figure 1E,F), while the aryl hydrocarbon pathway is weakly inactivated. The inhibition of the matrix metalloproteinases pathway has an equal chance to be activated or inactivated by UV‐only (Figure 1E). In both UV‐only and complete solar conditions, the GADD45 pathway shows clear activation following irradiation, while WNT/β‐catenin and wound healing show either clear activation or inhibition depending on the condition; WNT/β‐catenin is activated in the presence of UV, while wound healing is activated by UV light independently. For some pathways, there is no directionality that can be predicted, as shown by gray bars. A variety of pathways encompassing effects on the cell cycle, inflammation and wound healing, and the ECM were chosen for further analysis.

A total of 8 genes were chosen for further analysis from a variety of pathways found in Figure 1. The DEG lists for a specific pathway were similar between conditions, but not the same. Although the same genes interacted with each other in the same pathway, not all genes were differentially expressed in each condition. Most of the genes selected were involved in multiple pathways that were of interest. The pathways and genes chosen for further analysis are shown in Table 2.

**TABLE 2:** Pathways and genes relevant to skin aging chosen for qPCR analysis. Pathways and genes were identified using Ingenuity Pathway Analysis (IPA) software.

Pathway	*p*‐value (−log_10_)		Genes of Interest
	UV‐only	Complete Solar	
Aryl hydrocarbon receptor signaling pathway	5.85	4.76	CCNE2 CDKN1A MDM2
Cell cycle G1/S checkpoint regulation pathway	2.75	3.83	CCNE2 CDKN1A MDM2 SMAD3
GADD45 pathway	1.85	2.26	CCNE2 CDKN1A SMAD3
Inhibition of matrix metalloproteinases pathway	4.47	4.21	MMP1 MMP3
P53 pathway	5.38	4.98	CDKN1A MDM2
Prostanoid biosynthesis pathway	2.44	2.34	PTGS1 PTGES
RAR activation pathway	2.89	2.15	MMP1
UVA‐induced MAPK pathway	1.69	1.61	MDM2
Wound healing pathway	2.34	2.70	MMP1

### 3.2 Inorganic UV Filters Reduce MMP Production

**FIGURE 2:**
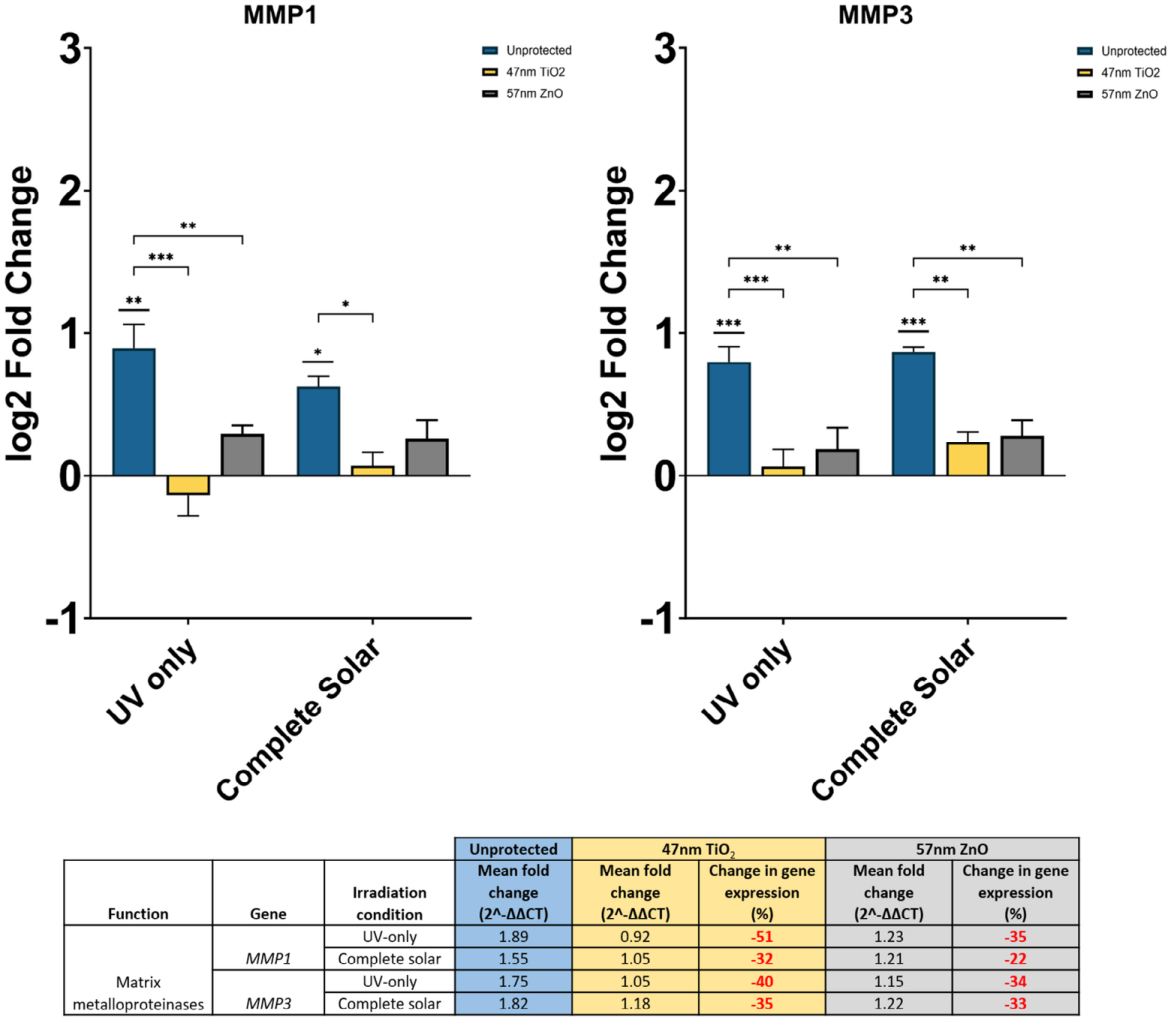
Gene expression of MMP1 and MMP3 in neonatal human dermal fibroblasts, 24 h after irradiation with UV‐only and complete solar light. Photoprotection, or lack of, is denoted by the color of bars as shown in the legend. Both filters are at 25% active level. Fold changes were calculated using the ∆∆CT method and converted to log_2_ values in the graphs. Percentage differences in gene expression for unprotected against protected fibroblasts are shown in the table: Red % change = reduction in expression. Expression assayed with TaqMan qPCR. Data represents mean ± SEM, *n* = 3. *p < = 0.05, **p < = 0.01, ***p < = 0.001.

The inorganic UV filters minimized irradiation‐induced changes to MMP1 and MMP3 expression by 22%–51% (Figure 2). In unprotected fibroblasts, MMP1 was found to be significantly upregulated by both UV‐only and complete solar light (UV‐only *p* < = 0.01, complete solar *p* < = 0.05); in the complete solar condition, MMP1 was upregulated by almost twofold. Upon applying 57 nm ZnO protection, there was a 35% reduction in gene expression following UV‐only irradiation and a 22% reduction following complete solar irradiation. The directionality of MMP1 remained positive, and thus upregulation was maintained. On the contrary, photoprotection with 47 nm TiO_2_ reduced MMP1 expression by 51% and 32% following UV‐only and complete solar irradiation, respectively. In the UV‐only condition, 47 nm TiO_2_ promoted downregulation of MMP1. Comparing the MMP1 gene expressions for unprotected and protected fibroblasts, both dispersions of inorganic UV filter produced a significant difference in the UV‐only condition (47 nm TiO_2_ *p* < = 0.001, 57 nm ZnO *p* < = 0.01).

MMP3 was significantly upregulated in unprotected fibroblasts following UV‐only and complete solar irradiation (*p* < = 0.001). MMP3 expression was minimized by 40% and 34% in the presence of 47 nm TiO_2_ and 57 nm ZnO, respectively, in the UV‐only condition. Alternatively, MMP3 expression was minimized by 35% and 33% in the complete solar condition, respectively. Both inorganic UV filters significantly reduced MMP3 expression in both irradiation conditions (*p* < = 0.01). Directionality was maintained in both irradiation conditions with either UV filter; MMP3 remained upregulated.

There were no significant differences in gene expression between the two UV filters (*p* > 0.05). Significance can only be realized when comparing the unprotected condition to either of the UV filters.

### 3.3 Inorganic UV Filters Significantly Reduce PTGES Expression

**FIGURE 3:**
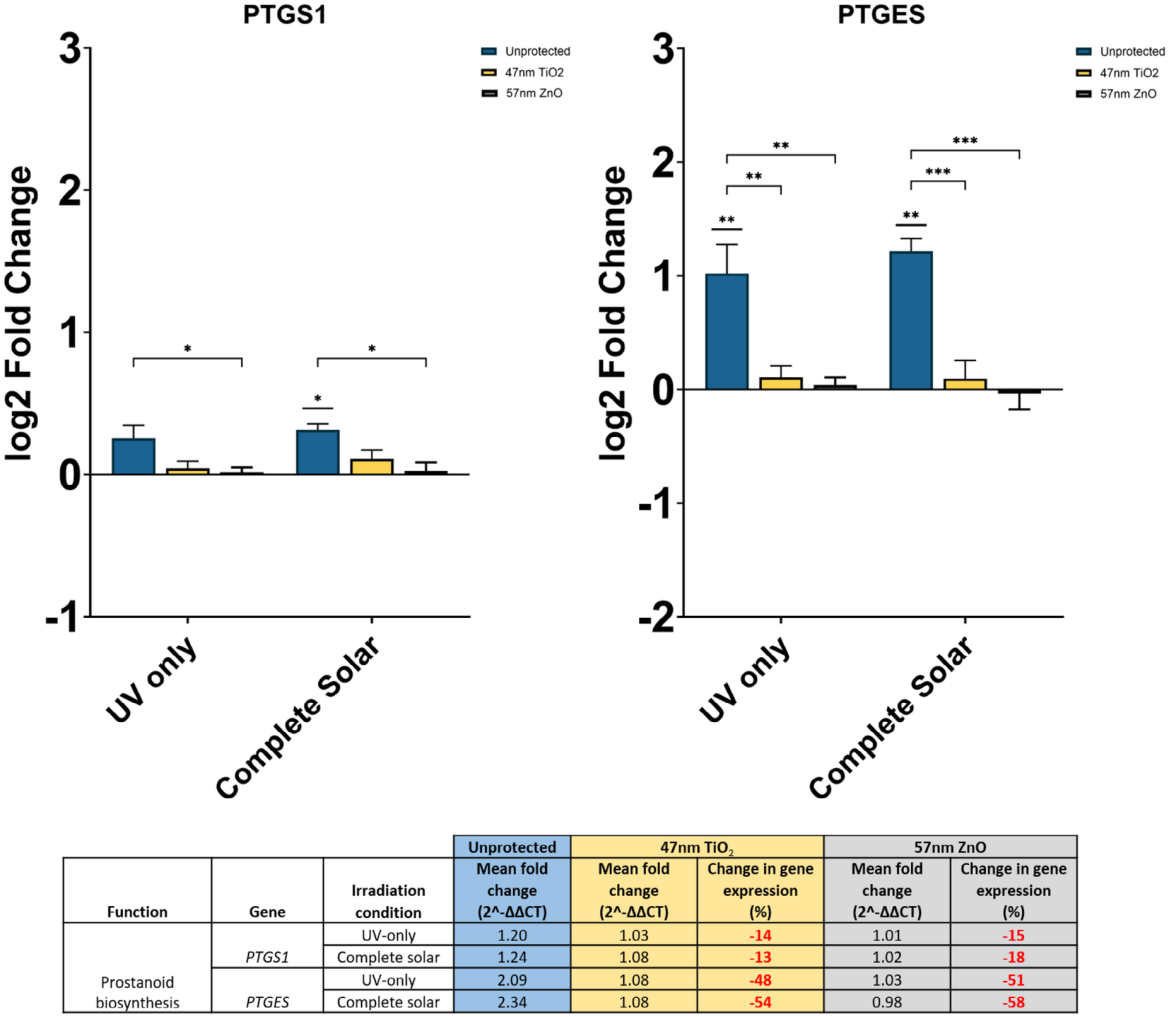
Gene expression of PTGS1 and PTGES in neonatal human dermal fibroblasts, 24 h after irradiation with UV‐only and complete solar light. Photoprotection, or lack of, is denoted by the color of bars as shown in the legend. Both filters are at 25% active level. Fold changes were calculated using the ∆∆CT method and converted to log_2_ values in the graphs. Percentage differences in gene expression for unprotected against protected fibroblasts are shown in the table: Red % change = reduction in expression. Expression assayed with TaqMan qPCR. Data represents mean ± SEM, *n* = 3. *p < = 0.05, **p < = 0.01, ***p < = 0.001.

Irradiation‐induced changes in the expression of prostaglandin biosynthesis genes, PTGS1 and PTGES, were minimized upon application of inorganic UV filters (Figure 3). In unprotected fibroblasts, both genes were upregulated, although to varying extents. PTGS1 did not exhibit a significant change in gene expression following UV‐only irradiation, and only a low significance was observed in the complete solar condition (*p* < = 0.05). On the contrary, PTGES was upregulated > 2 fold (log_2_ fold change > 1) in both irradiation conditions (*p* < = 0.01). Following protection with 47 nm TiO_2_ and 57 nm ZnO, upregulation of PTGS1 and PTGES was minimized to < 1.25 fold (log_2_ fold change < 0.3), regardless of UV filter or irradiation condition. Expression in protected fibroblasts had no significant difference against a non‐irradiated control.

PTGS1 expression following UV‐only irradiation was reduced by 14% and 15% in the presence of 47 nm TiO_2_ and 57 nm ZnO, respectively. In the complete solar light condition, PTGS1 was reduced by 13% and 18%, respectively. 57 nm ZnO induced a significant difference in PTGS1 expression against unprotected fibroblasts (*p* < = 0.05). Upregulation was maintained with both filters.

PTGES expression following UV‐only irradiation was reduced by 48% and 51% in the presence of 47 nm TiO_2_ and 57 nm ZnO, respectively. In the complete solar light condition, PTGES was reduced by 54% and 58%, respectively. Unlike PTGS1, there was a consistent significant difference observed in gene expression between unprotected and protected fibroblasts, regardless of UV filter and irradiation condition (UV‐only *p* < = 0.01, Complete solar *p* < = 0.001). Upregulation was maintained in both irradiation conditions with 47 nm TiO_2_. However, downregulation was observed for 57 nm ZnO in the complete solar condition.

There were no significant differences in gene expression between the two UV filters (*p* > 0.05). Significance can only be realized when comparing the unprotected condition to either of the UV filters.

### 3.4 Inorganic UV Filters Significantly Reduce Irradiation‐Induced Changes in Cell Cycle‐Related Genes

**FIGURE 4:**
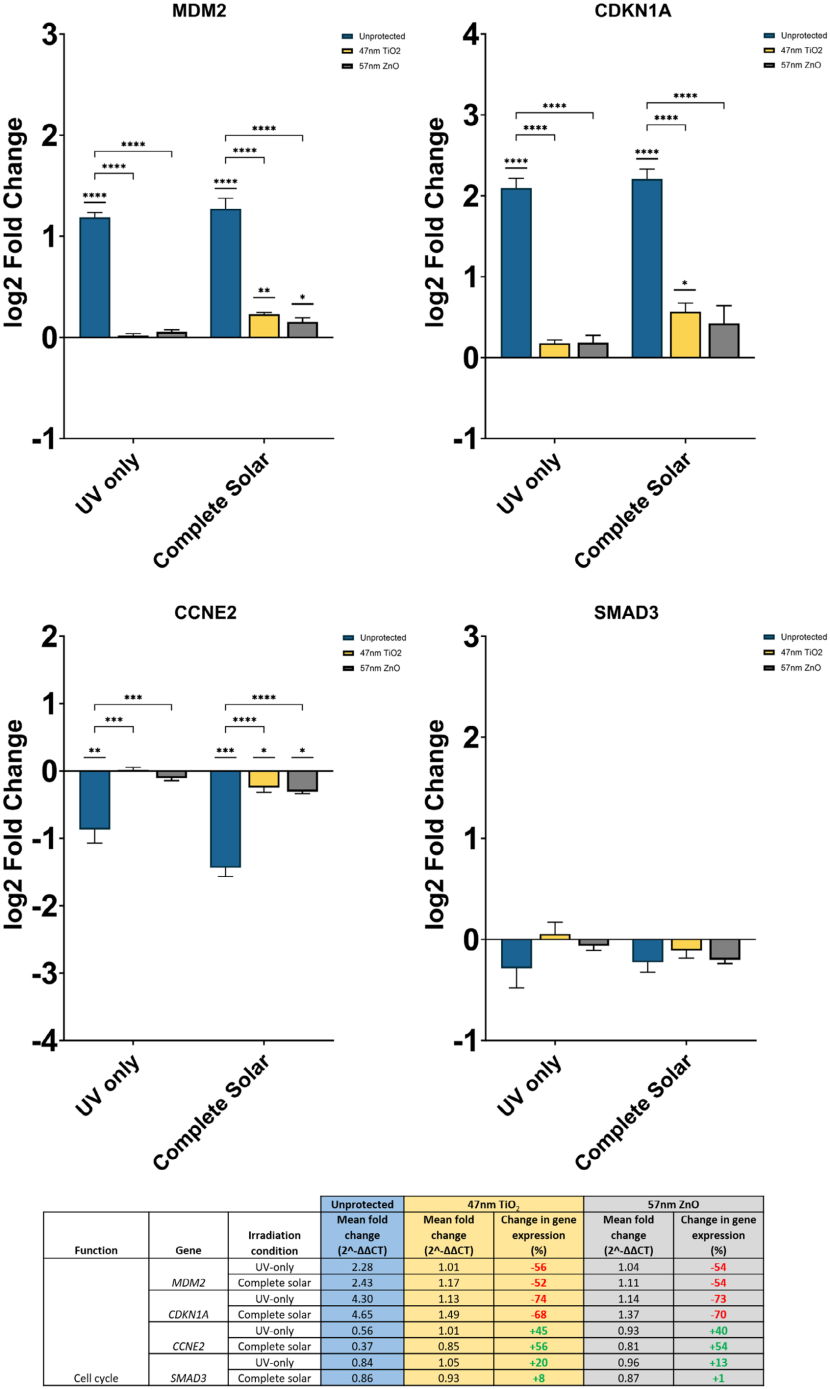
Gene expression of MDM2, CDKN1A, CCNE2, and SMAD3 in neonatal human dermal fibroblasts, 24 h after irradiation with UV‐only and complete solar light. Photoprotection, or lack of, is denoted by the color of bars as shown in the legend. Both filters are at 25% active level. Fold changes were calculated using the ∆∆CT method and converted to log_2_ values in the graphs. Percentage differences in gene expression for unprotected against protected fibroblasts are shown in the table: Red % change = reduction in expression, green % change = increase in expression. Expression assayed with TaqMan qPCR. Data represents mean ± SEM, *n* = 3. *p < = 0.05, **p < = 0.01, ***p < = 0.001, ****p < = 0.0001.

Irradiation‐induced changes in three of the four cell cycle genes investigated were significantly alleviated in the presence of inorganic UV filters (Figure 4). Upregulation of MDM2 and CDKN1A, as well as downregulation of CCNE2, caused by UV‐only and complete solar irradiation, were all significantly minimized upon protection with 47 nm TiO_2_ and 57 nm ZnO. In particular, MDM2 and CDKN1A exhibited the greatest difference in fold change between unprotected and protected fibroblasts amongst all 8 marker genes investigated. The significance in difference was indiscriminate of the type of UV filter used and the irradiation condition (*p* < = 0.0001).

In unprotected fibroblasts, MDM2 was upregulated by over twofold in both irradiation conditions (log_2_ fold change > 1) (*p* < = 0.0001) whilst CDKN1A was upregulated by over fourfold in both irradiation conditions (log_2_ fold change > 2) (*p* < = 0.0001). Upregulation of MDM2 was minimized by 54%–56% in the UV‐only condition and 52%–54% in the complete solar condition upon application of inorganic UV filters. Despite a significant reduction in upregulation against unprotected fibroblasts, the fold changes for MDM2 with 47 nm TiO_2_ and 57 nm ZnO photoprotection remained significantly different against a non‐irradiated control following complete solar irradiation (47 nm TiO_2_ *p* < = 0.01, 57 nm ZnO *p* < = 0.05). Meanwhile, upregulation of CDKN1A was minimized by 73%–74% in the UV‐only condition and 68%–70% in the complete solar condition, with inorganic filters. CDKN1A expression with 47 nm TiO_2_ photoprotection remained significantly different against a non‐irradiated control following complete solar irradiation (*p* < = 0.05).

For both genes, the directionality of gene expression remained constant with inorganic filter protection in both irradiation conditions. Regardless, there were no significant differences in gene expression between the two UV filters (*p* > = 0.05). Significance can only be realized when comparing the unprotected condition to either of the two UV filters.

Unlike MDM2 and CDKN1A, CCNE2 was significantly downregulated in unprotected fibroblasts upon irradiation with UV‐only (*p* < = 0.01) and complete solar light (*p* < = 0.001). The UV filters reduced the extent of downregulation, and an increase in gene expression was observed instead. In the UV‐only condition, CCNE2 expression increased by 45% and 40% following protection with 47 nm TiO_2_ and 57 nm ZnO, respectively. Likewise, in the complete solar condition, CCNE2 expression increased by 56% and 54%, respectively. CCNE2 expression in the complete solar condition remained significantly different against a non‐irradiated control with both UV filters (*p* < = 0.05). Downregulation of CCNE2 was conserved regardless of UV filter and irradiation condition. There were no significant differences in gene expression between the two UV filters (*p* > = 0.05).

Irradiation‐induced changes in the fourth cell cycle gene investigated, SMAD3, were reduced by the inorganic filters, but no significant difference was observed between protected and unprotected fibroblasts. The gene expression of SMAD3 also held no difference against a non‐irradiated control, regardless of UV filter and irradiation condition. Generally, downregulation is the observed directionality for SMAD3; however, 47 nm TiO_2_ may induce upregulation in the UV‐only condition.

## 4 Discussion

### 4.1 UV and Visible Light Affect the Cell Cycle at Different Checkpoints

A variety of cell‐cycle related pathways were activated by complete solar and UV‐only irradiation; the most notable are the p53 signaling pathway, cell cycle G1/S checkpoint regulation pathway, and GADD45 pathway (Figure 1). Activation of the G1/S checkpoint regulation pathway indicates early cell cycle arrest, which is linked to the activation of the p53 tumor suppressor gene following UV‐induced DNA damage [31]. This stipulates a link between multiple cell cycle‐related pathways. Additionally, activation of GADD45 suggests the production of DNA‐repairing GADD45 proteins, which have been found to cause downstream cell arrest at the G2/M phase of the cell cycle in fibroblasts [24, 32].

Three of the four cell‐cycle related genes chosen for qPCR analysis (CCNE2, CDKN1A, MDM2) were all shared between two or more of the cell‐cycle pathways investigated and were significantly upregulated or downregulated following irradiation without photoprotection (Figure 4). Cyclin E2 (CCNE2) is one of two E‐cyclins that bind with cyclin‐dependent kinase 2 (CDK2) to promote the G1/S transition of the cell cycle [33]. Downregulation of CCNE2 in both RNAseq and qPCR data indicates that irradiation with UV causes cell cycle arrest at the G1/S phase. Likewise, cyclin‐dependent kinase inhibitor 1A (CDKN1A) encodes for an inhibitor protein which also promotes G1/S arrest; in the RNAseq and qPCR data, this gene is upregulated in all conditions. Lastly, mouse double minute 2 (MDM2) is a negative regulator of the p53 tumor suppressor gene and is upregulated when p53 activity is increased. Upon photoprotection with TiO_2_ or ZnO nanoparticles, the three cell cycle genes above experienced the most significant reductions in irradiation‐induced expression changes, by 40%–75%. Thus, the impact of these inorganic filters are more pronounced in preventing the activation of p53, cell cycle arrest at the G1/S phase, and GADD45 production, with little difference observed between filters.

### 4.2 UV and Visible Light Upregulates Matrix Metalloproteinase Activity

The inhibition of the matrix metalloproteinases pathway is another significant signaling pathway affected by UV‐related irradiation. Matrix metalloproteinases (MMPs) are enzymes that break down the structural components of the ECM and play a significant role in the maintenance of the dermis. Both the RNAseq and qPCR results show that MMP1 and MMP3 are upregulated following UV‐related irradiation, which concurs with previous studies where UV causes increased levels of several MMPs [34, 35, 36]. Both UVA and UVB‐mediated production of ROS contribute to the upregulation of MMPs by activating activator protein‐1 (AP‐1) through MAPK signaling [37, 38]. The z‐score observed in the RNAseq data (Figure 1) correlates with literature, as inhibition of this pathway means that MMP production is induced. MMPs target different components of the ECM such as collagens, elastins, and fibrins, which can lead to fragmentation or loss of the structural proteins [39, 40]; this can cause skin to exhibit photoaged characteristics such as wrinkling and loss of elasticity [38]. Both inorganic filters applied caused a reduction in MMP1 and MMP3 upregulation by 22%–51% and ~35.5%, respectively (Figure 2). Reduction in MMP3 expression was more consistent than MMP1, regardless of filter type or irradiation condition, and exhibited a consistent significant difference.

### 4.3 UV and Visible Light Can Influence the Prostanoid Biosynthesis Pathway

Prostanoid biosynthesis is a pathway that is affected by UV irradiation but does not show clear activation or inhibition based on the z‐score (Figure 1). It is an important pathway to consider in dermatology as it leads to the production of prostaglandins, which have a role in inflammation. Prostaglandin E2 (PGE2) is the most notable prostaglandin and has been linked to the onset of skin reddening and swelling due to its effect on arterial dilation in vivo [41]. Alternatively, PGE2 has been found to inhibit collagen production and increase MMP1 expression in human dermal fibroblasts in vitro; these are two conditions that accelerate skin aging [42].

The DEGs identified in this pathway which underwent qPCR analysis were prostaglandin G/H synthase‐1 (PTGS1) and prostaglandin E synthase (PTGES). PTGS1, also known as cyclooxygenase‐1, is an enzyme which catalyzes the conversion of arachidonic acid (AA) into prostaglandin while PTGES catalyzes the conversion of prostaglandin endoperoxide H2 (PGH2) into PGE2 during inflammation [43]. Both genes must be activated for the downstream synthesis of PGE2 which impacts dermal vasculature in vivo and the ECM of the dermis in vitro [41, 42]. Although both genes were classed as DEGs in the RNAseq, only PTGES showed a significant difference in expression with both irradiation conditions in qPCR (Figure 3). Additionally, downregulation of PTGES was observed, which displays opposite directionality to RNAseq. This may be a case of PTGES being a non‐concordant gene, which constitutes approximately 15%–20% of genes compared between RNAseq and qPCR [44]. Regardless, the significance of expression indicates the impact of UV on terminal prostaglandin production. Irradiation‐induced upregulation of both prostaglandin genes was minimized by the two inorganic filters by < 20% for PTGS1 and ~52.75% for PTGES. The differences suggest that TiO_2_ and ZnO can minimize prostaglandin‐mediated inflammation by targeting terminal prostaglandin production.

### 4.4 Scientific and Cosmetic Relevance of Study and Future Adaptations

The approach taken in this study serves as a foundation for high throughput qPCR studies, concerned with different pathways associated with photoaging and understanding the photoprotective effects of different UV filters at a genetic level. This study focused solely on dermal fibroblasts, which uphold the structure and elasticity of skin as the key cell type of the dermal layer susceptible to UV‐induced photodamage. However, future studies could implement and adapt the current approach taken by considering the use of keratinocytes or melanocytes, as both cell types influence skin aging phenotypes in the epidermal layer. Alternatively, the use of 3D skin tissue models, which integrate all three cell types, would prove to be a more accurate representation of UV irradiation response, taking into account interactions between the dermis and epidermis.

A parameter that could also be suitably adjusted in future studies would be response time to irradiation; 24 h post‐irradiation was observed in this study as it was the minimum time to exhibit an observable effect of irradiation, but photodamage can extend beyond this timeframe to 48,72, and 96 h. Additionally, the data acquired can be validated through protein studies, using western blot or ELISA assays to understand whether there is a correlation between photoaging‐related marker genes and resultant proteins. The vast information that could be obtained would assist cosmetic manufacturers in optimizing sunscreen formulations by visualizing the efficacy of different inorganic active ingredients at various active levels in targeting irradiation‐induced signaling pathways related to photoaging.

## 5 Conclusion

Titanium dioxide and zinc oxide‐based inorganic UV filters < 100 nm have shown to be effective in alleviating irradiation‐induced upregulation or downregulation with regard to certain cell cycle, prostanoid biosynthesis, and matrix metalloproteinase‐related genes. In turn, this could influence the activation or inhibition of signaling pathways that contribute to photoaging. Although no significant difference was observed to discriminate the advantage of each UV filter at the genetic level, expression of genes related to MMP production, inflammation, and cell cycle arrest were maintained close to non‐irradiated levels with both UV filters. Our study provides positive novel insights into how inorganic‐based photoprotection has an anti‐photoaging impact on cell signaling and gene activity in dermal fibroblasts.

## Author Contributions

Mark A. Birch‐Machin, as the senior and corresponding author, co‐designed the research project with Bhaven Chavan. Neil Dominic T. Pangilinan performed the experiments based on preliminary work by Catherine Bonn. Neil Dominic T. Pangilinan wrote the paper. Mohammad Shalbaf and Aline Souza supervised the project as representatives of Croda Europe ltd. and provided the materials for producing the inorganic dispersions. Mark A. Birch‐Machin, Bhaven Chavan, Mohammad Shalbaf, and Aline Souza provided comments on the manuscript.

## Conflicts of Interest

The authors declare no conflicts of interest.

## Data Availability

The data that support the findings of this study are available on request from the corresponding author. The data are not publicly available due to privacy or ethical restrictions.
